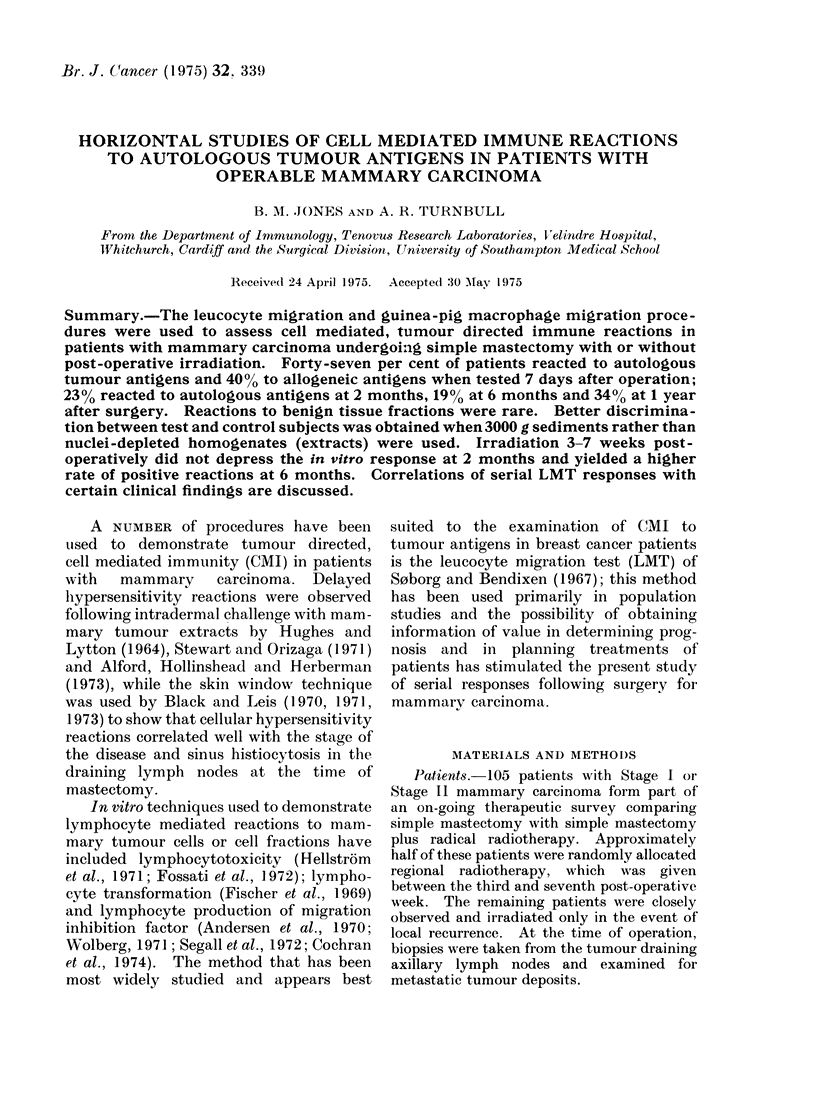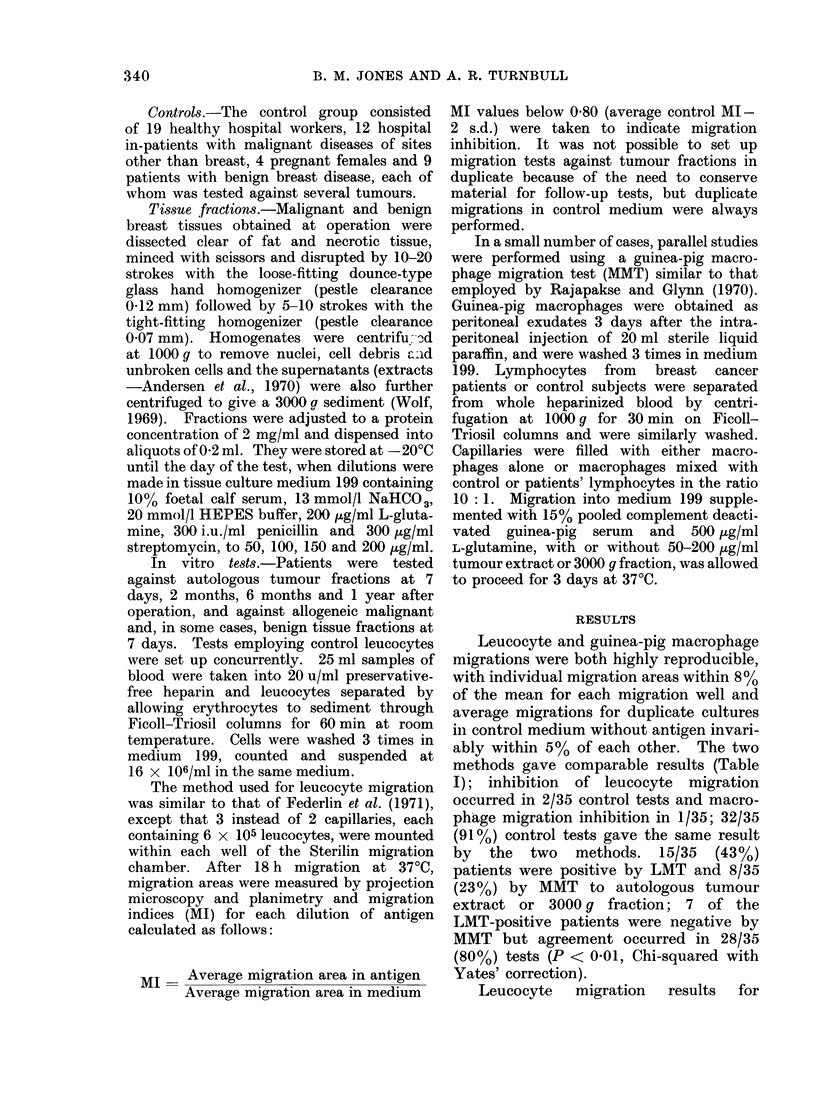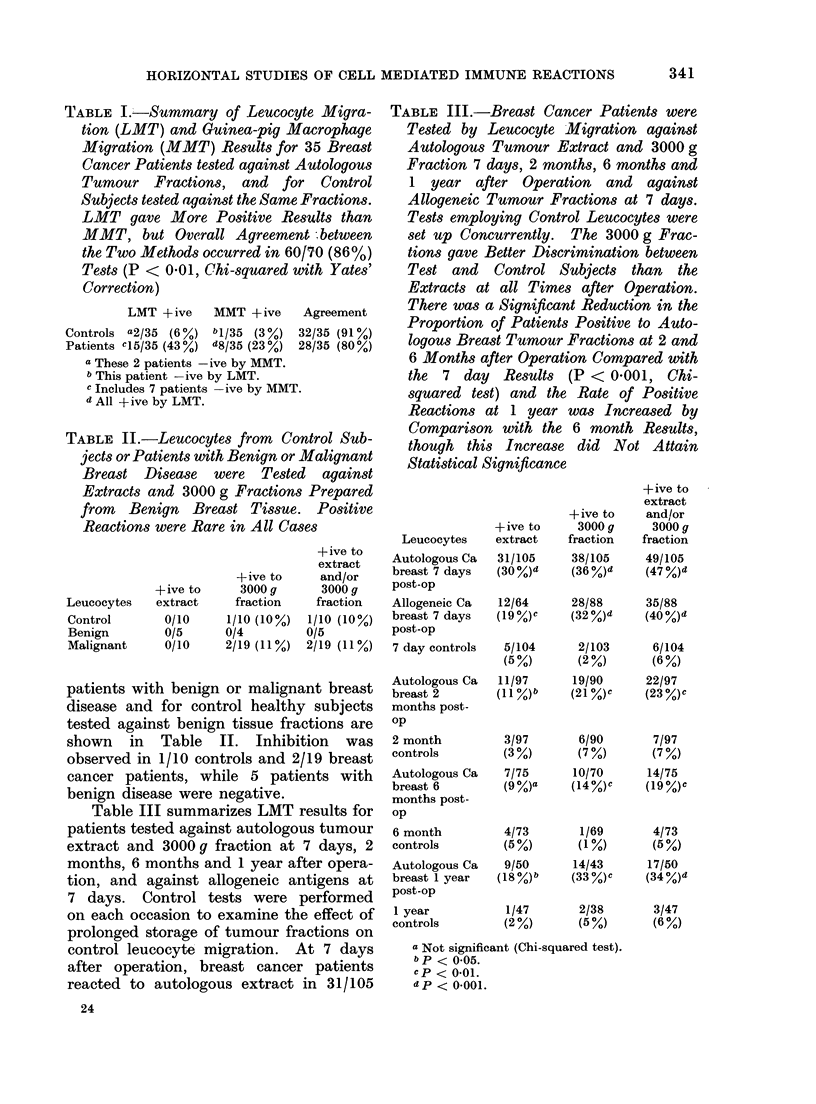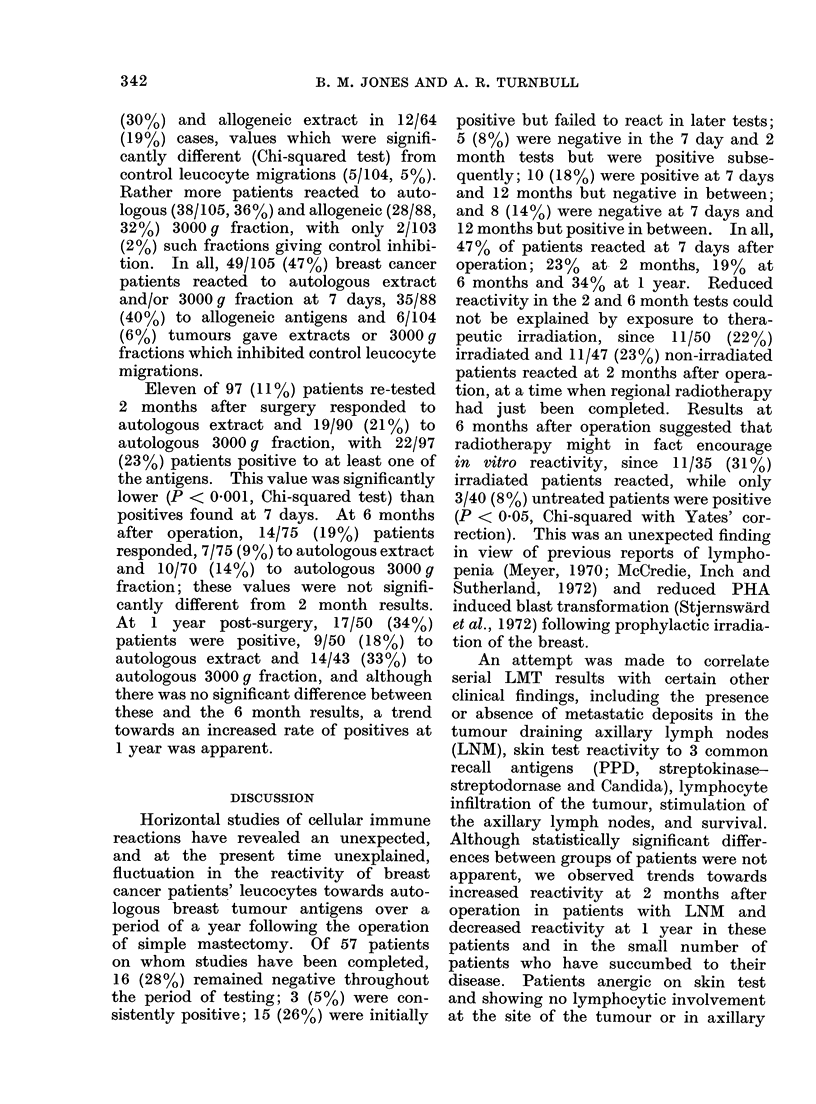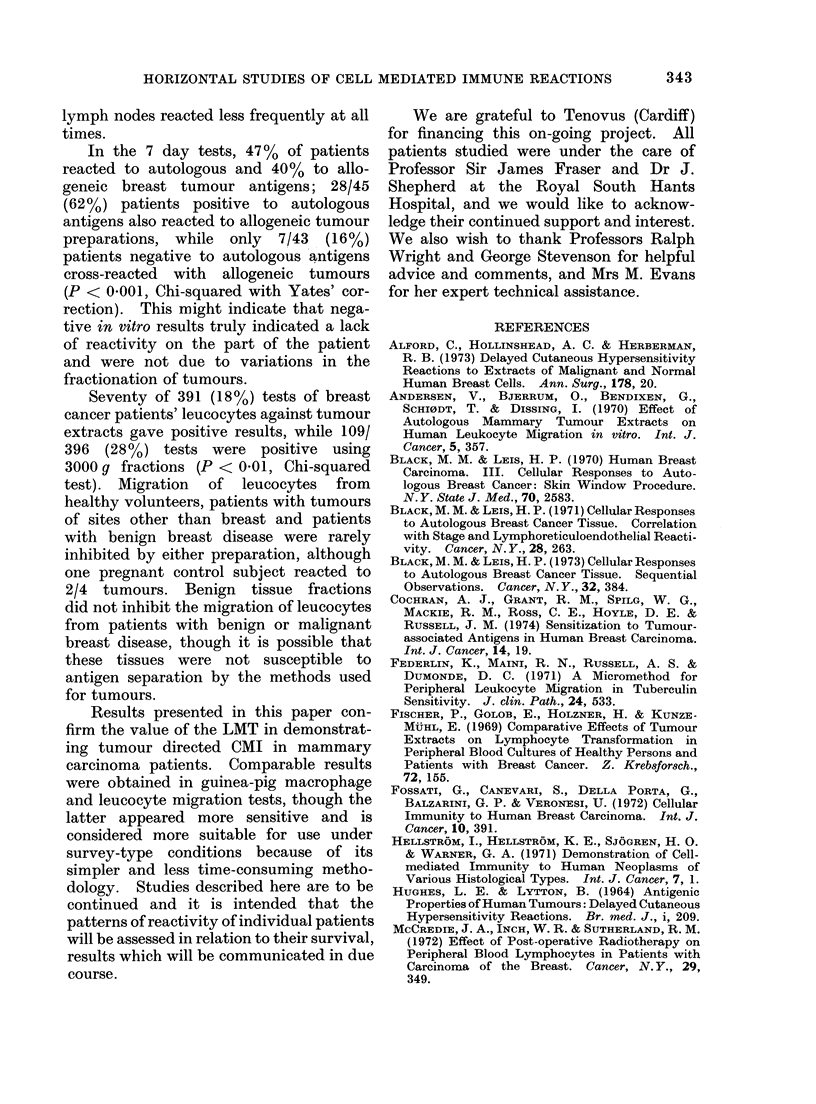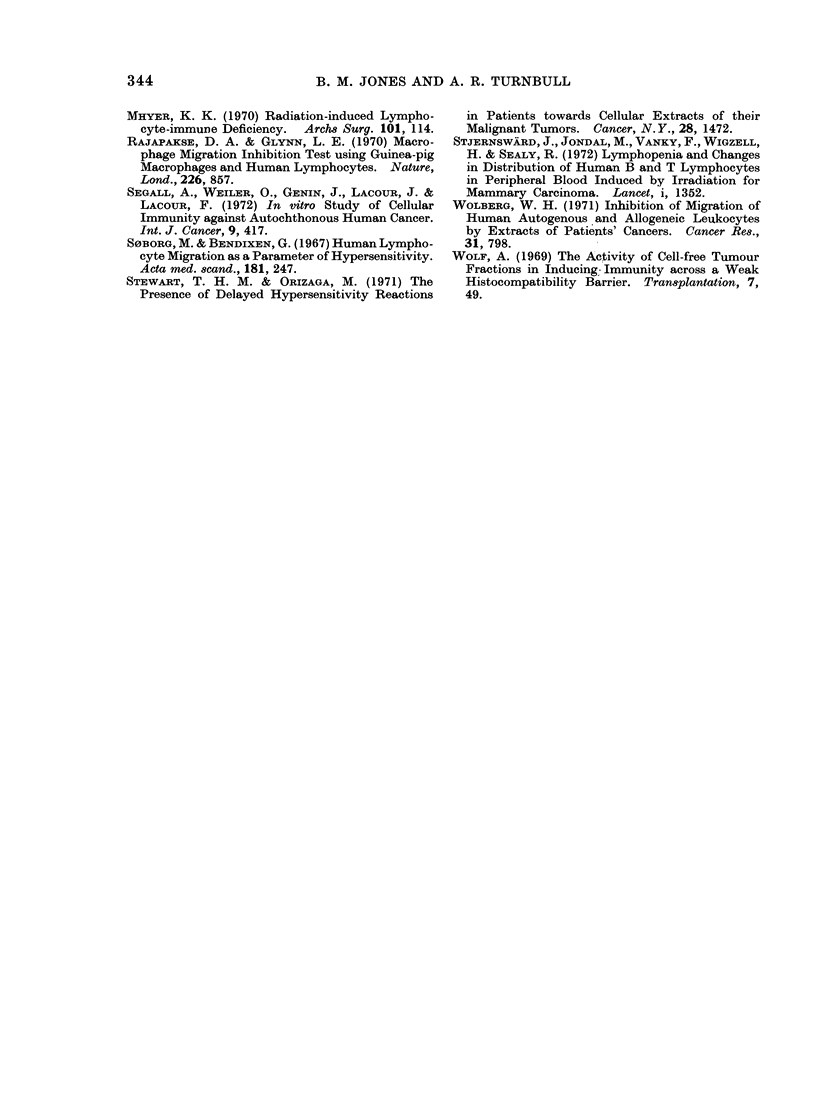# Horizontal studies of cell mediated immune reactions to autologous tumour antigens in patients with operable mammary carcinoma.

**DOI:** 10.1038/bjc.1975.232

**Published:** 1975-09

**Authors:** B. M. Jones, A. R. Turnbull

## Abstract

The leucocyte migration and guinea-pig macrophage migration procedures were used to assess cell mediated, tumour directed immune reactions in patients with mammary carcinoma undergoing simple mastectomy with or without post-operative irradiation. Forty-seven per cent of patients reacted to autologous tumour antigens and 40% to allogeneic antigens when tested 7 days after operation; 23% reacted to autologous antigens at 2 months, 19% at 6 months and 34% at 1 year after surgery. Reactions to benign tissue fractions were rare. Better discrimination between test and control subjects was obtained when 3000 g sediments rather than nuclei-depleted homogenates (extracts) were used. Irradiation 3-7 weeks post-operatively did not depress the in vitro response at 2 months and yielded a higher rate of positive reactions at 6 months. Correlations of serial LMT responses with certain clinical findings are discussed.


*Br. J. Cancer* (1975) **32**, 339

### HORIZONTAL STUDIES OF CELL MEDIATED IMMUNE REACTIONS TO AUTOLOGOUS TUMOUR ANTIGENS IN PATIENTS WITH OPERABLE MAMMARY CARCINOMA

B. M. JONES AND A. R. TURNBULL

*From the Department of Immunology, Tenovus Research Laboratories, Velindre Hospital, Whitchurch, Cardiff and the Surgical Division, University of Southampton Medical School*

Received 24 April 1975. Accepted 30 May 1975

**Summary.—The leucocyte migration and guinea-pig macrophage migration procedures were used to assess cell mediated, tumour directed immune reactions in patients with mammary carcinoma undergoing simple mastectomy with or without post-operative irradiation. Forty-seven per cent of patients reacted to autologous tumour antigens and 40% to allogeneic antigens when tested 7 days after operation; 23% reacted to autologous antigens at 2 months, 19% at 6 months and 34% at 1 year after surgery. Reactions to benign tissue fractions were rare. Better discrimination between test and control subjects was obtained when 3000 *g* sediments rather than nuclei-depleted homogenates (extracts) were used. Irradiation 3–7 weeks post-operatively did not depress the *in vitro* response at 2 months and yielded a higher rate of positive reactions at 6 months. Correlations of serial LMT responses with certain clinical findings are discussed.**

A NUMBER of procedures have been used to demonstrate tumour directed, cell mediated immunity (CMI) in patients with mammary carcinoma. Delayed hypersensitivity reactions were observed following intradermal challenge with mammary tumour extracts by Hughes and Lytton (1964), Stewart and Orizaga (1971) and Alford, Hollinshead and Herberman (1973), while the skin window technique was used by Black and Leis (1970, 1971, 1973) to show that cellular hypersensitivity reactions correlated well with the stage of the disease and sinus histiocytosis in the draining lymph nodes at the time of mastectomy.

*In vitro* techniques used to demonstrate lymphocyte mediated reactions to mammary tumour cells or cell fractions have included lymphocytotoxicity (Hellström *et al.*, 1971; Fossati *et al.*, 1972); lymphocyte transformation (Fischer *et al.*, 1969) and lymphocyte production of migration inhibition factor (Andersen *et al.*, 1970; Wolberg, 1971; Segall *et al.*, 1972; Cochran *et al.*, 1974). The method that has been most widely studied and appears best suited to the examination of CMI to tumour antigens in breast cancer patients is the leucocyte migration test (LMT) of Søborg and Bendixen (1967); this method has been used primarily in population studies and the possibility of obtaining information of value in determining prognosis and in planning treatments of patients has stimulated the present study of serial responses following surgery for mammary carcinoma.

### MATERIALS AND METHODS

*Patients.*—105 patients with Stage I or Stage II mammary carcinoma form part of an on-going therapeutic survey comparing simple mastectomy with simple mastectomy plus radical radiotherapy. Approximately half of these patients were randomly allocated regional radiotherapy, which was given between the third and seventh post-operative week. The remaining patients were closely observed and irradiated only in the event of local recurrence. At the time of operation, biopsies were taken from the tumour draining axillary lymph nodes and examined for metastatic tumour deposits.

*Controls*.—The control group consisted of 19 healthy hospital workers, 12 hospital in-patients with malignant diseases of sites other than breast, 4 pregnant females and 9 patients with benign breast disease, each of whom was tested against several tumours.

*Tissue fractions*.—Malignant and benign breast tissues obtained at operation were dissected clear of fat and necrotic tissue, minced with scissors and disrupted by 10–20 strokes with the loose-fitting dounce-type glass hand homogenizer (pestle clearance 0·12 mm) followed by 5–10 strokes with the tight-fitting homogenizer (pestle clearance 0·07 mm). Homogenates were centrifuged at 1000 *g* to remove nuclei, cell debris and unbroken cells and the supernatants (extracts —Andersen *et al.*, 1970) were also further centrifuged to give a 3000 *g* sediment (Wolf, 1969). Fractions were adjusted to a protein concentration of 2 mg/ml and dispensed into aliquots of 0·2 ml. They were stored at −20°C until the day of the test, when dilutions were made in tissue culture medium 199 containing 10% foetal calf serum, 13 mmol/l $NaHCO_3$, 20 mmol/l HEPES buffer, 200 μg/ml L-glutamine, 300 i.u./ml penicillin and 300 μg/ml streptomycin, to 50, 100, 150 and 200 μg/ml.

*In vitro tests*.—Patients were tested against autologous tumour fractions at 7 days, 2 months, 6 months and 1 year after operation, and against allogeneic malignant and, in some cases, benign tissue fractions at 7 days. Tests employing control leucocytes were set up concurrently. 25 ml samples of blood were taken into 20 u/ml preservative-free heparin and leucocytes separated by allowing erythrocytes to sediment through Ficoll–Triosil columns for 60 min at room temperature. Cells were washed 3 times in medium 199, counted and suspended at $16 \times 10^6$/ml in the same medium.

The method used for leucocyte migration was similar to that of Federlin *et al.* (1971), except that 3 instead of 2 capillaries, each containing $6 \times 10^5$ leucocytes, were mounted within each well of the Sterilin migration chamber. After 18 h migration at 37°C, migration areas were measured by projection microscopy and planimetry and migration indices (MI) for each dilution of antigen calculated as follows:

$$\mathrm{MI} = \frac{\text{Average migration area in antigen}}{\text{Average migration area in medium}}$$

MI values below 0·80 (average control MI − 2 s.d.) were taken to indicate migration inhibition. It was not possible to set up migration tests against tumour fractions in duplicate because of the need to conserve material for follow-up tests, but duplicate migrations in control medium were always performed.

In a small number of cases, parallel studies were performed using a guinea-pig macrophage migration test (MMT) similar to that employed by Rajapakse and Glynn (1970). Guinea-pig macrophages were obtained as peritoneal exudates 3 days after the intraperitoneal injection of 20 ml sterile liquid paraffin, and were washed 3 times in medium 199. Lymphocytes from breast cancer patients or control subjects were separated from whole heparinized blood by centrifugation at 1000 *g* for 30 min on Ficoll–Triosil columns and were similarly washed. Capillaries were filled with either macrophages alone or macrophages mixed with control or patients' lymphocytes in the ratio 10 : 1. Migration into medium 199 supplemented with 15% pooled complement deactivated guinea-pig serum and 500 μg/ml L-glutamine, with or without 50–200 μg/ml tumour extract or 3000 *g* fraction, was allowed to proceed for 3 days at 37°C.

### RESULTS

Leucocyte and guinea-pig macrophage migrations were both highly reproducible, with individual migration areas within 8% of the mean for each migration well and average migrations for duplicate cultures in control medium without antigen invariably within 5% of each other. The two methods gave comparable results (Table I); inhibition of leucocyte migration occurred in 2/35 control tests and macrophage migration inhibition in 1/35; 32/35 (91%) control tests gave the same result by the two methods. 15/35 (43%) patients were positive by LMT and 8/35 (23%) by MMT to autologous tumour extract or 3000 *g* fraction; 7 of the LMT-positive patients were negative by MMT but agreement occurred in 28/35 (80%) tests ($P < 0{\cdot}01$, Chi-squared with Yates' correction).

Leucocyte migration results for

TABLE I.—*Summary of Leucocyte Migration (LMT) and Guinea-pig Macrophage Migration (MMT) Results for 35 Breast Cancer Patients tested against Autologous Tumour Fractions, and for Control Subjects tested against the Same Fractions. LMT gave More Positive Results than MMT, but Overall Agreement between the Two Methods occurred in 60/70 (86%) Tests (P < 0·01, Chi-squared with Yates' Correction)*

| | LMT +ive | MMT +ive | Agreement |
|---|---|---|---|
| Controls | [a]2/35 (6%) | [b]1/35 (3%) | 32/35 (91%) |
| Patients | [c]15/35 (43%) | [d]8/35 (23%) | 28/35 (80%) |

[a] These 2 patients −ive by MMT.
[b] This patient −ive by LMT.
[c] Includes 7 patients −ive by MMT.
[d] All +ive by LMT.

TABLE II.—*Leucocytes from Control Subjects or Patients with Benign or Malignant Breast Disease were Tested against Extracts and 3000 g Fractions Prepared from Benign Breast Tissue. Positive Reactions were Rare in All Cases*

| Leucocytes | +ive to extract | +ive to 3000 *g* fraction | +ive to extract and/or 3000 *g* fraction |
|---|---|---|---|
| Control | 0/10 | 1/10 (10%) | 1/10 (10%) |
| Benign | 0/5 | 0/4 | 0/5 |
| Malignant | 0/10 | 2/19 (11%) | 2/19 (11%) |

patients with benign or malignant breast disease and for control healthy subjects tested against benign tissue fractions are shown in Table II. Inhibition was observed in 1/10 controls and 2/19 breast cancer patients, while 5 patients with benign disease were negative.

Table III summarizes LMT results for patients tested against autologous tumour extract and 3000 *g* fraction at 7 days, 2 months, 6 months and 1 year after operation, and against allogeneic antigens at 7 days. Control tests were performed on each occasion to examine the effect of prolonged storage of tumour fractions on control leucocyte migration. At 7 days after operation, breast cancer patients reacted to autologous extract in 31/105

TABLE III.—*Breast Cancer Patients were Tested by Leucocyte Migration against Autologous Tumour Extract and 3000 g Fraction 7 days, 2 months, 6 months and 1 year after Operation and against Allogeneic Tumour Fractions at 7 days. Tests employing Control Leucocytes were set up Concurrently. The 3000 g Fractions gave Better Discrimination between Test and Control Subjects than the Extracts at all Times after Operation. There was a Significant Reduction in the Proportion of Patients Positive to Autologous Breast Tumour Fractions at 2 and 6 Months after Operation Compared with the 7 day Results (P < 0·001, Chi-squared test) and the Rate of Positive Reactions at 1 year was Increased by Comparison with the 6 month Results, though this Increase did Not Attain Statistical Significance*

| Leucocytes | +ive to extract | +ive to 3000 *g* fraction | +ive to extract and/or 3000 *g* fraction |
|---|---|---|---|
| Autologous Ca breast 7 days post-op | 31/105 (30%)[d] | 38/105 (36%)[d] | 49/105 (47%)[d] |
| Allogeneic Ca breast 7 days post-op | 12/64 (19%)[c] | 28/88 (32%)[d] | 35/88 (40%)[d] |
| 7 day controls | 5/104 (5%) | 2/103 (2%) | 6/104 (6%) |
| Autologous Ca breast 2 months post-op | 11/97 (11%)[b] | 19/90 (21%)[c] | 22/97 (23%)[c] |
| 2 month controls | 3/97 (3%) | 6/90 (7%) | 7/97 (7%) |
| Autologous Ca breast 6 months post-op | 7/75 (9%)[a] | 10/70 (14%)[c] | 14/75 (19%)[c] |
| 6 month controls | 4/73 (5%) | 1/69 (1%) | 4/73 (5%) |
| Autologous Ca breast 1 year post-op | 9/50 (18%)[b] | 14/43 (33%)[c] | 17/50 (34%)[d] |
| 1 year controls | 1/47 (2%) | 2/38 (5%) | 3/47 (6%) |

[a] Not significant (Chi-squared test).
[b] $P < 0{\cdot}05$.
[c] $P < 0{\cdot}01$.
[d] $P < 0{\cdot}001$.

(30%) and allogeneic extract in 12/64 (19%) cases, values which were significantly different (Chi-squared test) from control leucocyte migrations (5/104, 5%). Rather more patients reacted to autologous (38/105, 36%) and allogeneic (28/88, 32%) 3000 *g* fraction, with only 2/103 (2%) such fractions giving control inhibition. In all, 49/105 (47%) breast cancer patients reacted to autologous extract and/or 3000 *g* fraction at 7 days, 35/88 (40%) to allogeneic antigens and 6/104 (6%) tumours gave extracts or 3000 *g* fractions which inhibited control leucocyte migrations.

Eleven of 97 (11%) patients re-tested 2 months after surgery responded to autologous extract and 19/90 (21%) to autologous 3000 *g* fraction, with 22/97 (23%) patients positive to at least one of the antigens. This value was significantly lower ($P < 0{\cdot}001$, Chi-squared test) than positives found at 7 days. At 6 months after operation, 14/75 (19%) patients responded, 7/75 (9%) to autologous extract and 10/70 (14%) to autologous 3000 *g* fraction; these values were not significantly different from 2 month results. At 1 year post-surgery, 17/50 (34%) patients were positive, 9/50 (18%) to autologous extract and 14/43 (33%) to autologous 3000 *g* fraction, and although there was no significant difference between these and the 6 month results, a trend towards an increased rate of positives at 1 year was apparent.

### DISCUSSION

Horizontal studies of cellular immune reactions have revealed an unexpected, and at the present time unexplained, fluctuation in the reactivity of breast cancer patients' leucocytes towards autologous breast tumour antigens over a period of a year following the operation of simple mastectomy. Of 57 patients on whom studies have been completed, 16 (28%) remained negative throughout the period of testing; 3 (5%) were consistently positive; 15 (26%) were initially positive but failed to react in later tests; 5 (8%) were negative in the 7 day and 2 month tests but were positive subsequently; 10 (18%) were positive at 7 days and 12 months but negative in between; and 8 (14%) were negative at 7 days and 12 months but positive in between. In all, 47% of patients reacted at 7 days after operation; 23% at 2 months, 19% at 6 months and 34% at 1 year. Reduced reactivity in the 2 and 6 month tests could not be explained by exposure to therapeutic irradiation, since 11/50 (22%) irradiated and 11/47 (23%) non-irradiated patients reacted at 2 months after operation, at a time when regional radiotherapy had just been completed. Results at 6 months after operation suggested that radiotherapy might in fact encourage *in vitro* reactivity, since 11/35 (31%) irradiated patients reacted, while only 3/40 (8%) untreated patients were positive ($P < 0{\cdot}05$, Chi-squared with Yates' correction). This was an unexpected finding in view of previous reports of lymphopenia (Meyer, 1970; McCredie, Inch and Sutherland, 1972) and reduced PHA induced blast transformation (Stjernswärd *et al.*, 1972) following prophylactic irradiation of the breast.

An attempt was made to correlate serial LMT results with certain other clinical findings, including the presence or absence of metastatic deposits in the tumour draining axillary lymph nodes (LNM), skin test reactivity to 3 common recall antigens (PPD, streptokinase–streptodornase and Candida), lymphocyte infiltration of the tumour, stimulation of the axillary lymph nodes, and survival. Although statistically significant differences between groups of patients were not apparent, we observed trends towards increased reactivity at 2 months after operation in patients with LNM and decreased reactivity at 1 year in these patients and in the small number of patients who have succumbed to their disease. Patients anergic on skin test and showing no lymphocytic involvement at the site of the tumour or in axillary lymph nodes reacted less frequently at all times.

In the 7 day tests, 47% of patients reacted to autologous and 40% to allogeneic breast tumour antigens; 28/45 (62%) patients positive to autologous antigens also reacted to allogeneic tumour preparations, while only 7/43 (16%) patients negative to autologous antigens cross-reacted with allogeneic tumours ($P < 0{\cdot}001$, Chi-squared with Yates' correction). This might indicate that negative *in vitro* results truly indicated a lack of reactivity on the part of the patient and were not due to variations in the fractionation of tumours.

Seventy of 391 (18%) tests of breast cancer patients' leucocytes against tumour extracts gave positive results, while 109/396 (28%) tests were positive using 3000 *g* fractions ($P < 0{\cdot}01$, Chi-squared test). Migration of leucocytes from healthy volunteers, patients with tumours of sites other than breast and patients with benign breast disease were rarely inhibited by either preparation, although one pregnant control subject reacted to 2/4 tumours. Benign tissue fractions did not inhibit the migration of leucocytes from patients with benign or malignant breast disease, though it is possible that these tissues were not susceptible to antigen separation by the methods used for tumours.

Results presented in this paper confirm the value of the LMT in demonstrating tumour directed CMI in mammary carcinoma patients. Comparable results were obtained in guinea-pig macrophage and leucocyte migration tests, though the latter appeared more sensitive and is considered more suitable for use under survey-type conditions because of its simpler and less time-consuming methodology. Studies described here are to be continued and it is intended that the patterns of reactivity of individual patients will be assessed in relation to their survival, results which will be communicated in due course.

We are grateful to Tenovus (Cardiff) for financing this on-going project. All patients studied were under the care of Professor Sir James Fraser and Dr J. Shepherd at the Royal South Hants Hospital, and we would like to acknowledge their continued support and interest. We also wish to thank Professors Ralph Wright and George Stevenson for helpful advice and comments, and Mrs M. Evans for her expert technical assistance.

### REFERENCES

Alford, C., Hollinshead, A. C. & Herberman, R. B. (1973) Delayed Cutaneous Hypersensitivity Reactions to Extracts of Malignant and Normal Human Breast Cells. *Ann. Surg.*, **178**, 20.

Andersen, V., Bjerrum, O., Bendixen, G., Schiødt, T. & Dissing, I. (1970) Effect of Autologous Mammary Tumour Extracts on Human Leukocyte Migration *in vitro*. *Int. J. Cancer*, **5**, 357.

Black, M. M. & Leis, H. P. (1970) Human Breast Carcinoma. III. Cellular Responses to Autologous Breast Cancer: Skin Window Procedure. *N.Y. State J. Med.*, **70**, 2583.

Black, M. M. & Leis, H. P. (1971) Cellular Responses to Autologous Breast Cancer Tissue. Correlation with Stage and Lymphoreticuloendothelial Reactivity. *Cancer, N.Y.*, **28**, 263.

Black, M. M. & Leis, H. P. (1973) Cellular Responses to Autologous Breast Cancer Tissue. Sequential Observations. *Cancer, N.Y.*, **32**, 384.

Cochran, A. J., Grant, R. M., Spilg, W. G., Mackie, R. M., Ross, C. E., Hoyle, D. E. & Russell, J. M. (1974) Sensitization to Tumour-associated Antigens in Human Breast Carcinoma. *Int. J. Cancer*, **14**, 19.

Federlin, K., Maini, R. N., Russell, A. S. & Dumonde, D. C. (1971) A Micromethod for Peripheral Leukocyte Migration in Tuberculin Sensitivity. *J. clin. Path.*, **24**, 533.

Fischer, P., Golob, E., Holzner, H. & Kunze-Mühl, E. (1969) Comparative Effects of Tumour Extracts on Lymphocyte Transformation in Peripheral Blood Cultures of Healthy Persons and Patients with Breast Cancer. *Z. Krebsforsch.*, **72**, 155.

Fossati, G., Canevari, S., Della Porta, G., Balzarini, G. P. & Veronesi, U. (1972) Cellular Immunity to Human Breast Carcinoma. *Int. J. Cancer*, **10**, 391.

Hellström, I., Hellström, K. E., Sjögren, H. O. & Warner, G. A. (1971) Demonstration of Cell-mediated Immunity to Human Neoplasms of Various Histological Types. *Int. J. Cancer*, **7**, 1.

Hughes, L. E. & Lytton, B. (1964) Antigenic Properties of Human Tumours: Delayed Cutaneous Hypersensitivity Reactions. *Br. med. J.*, i, 209.

McCredie, J. A., Inch, W. R. & Sutherland, R. M. (1972) Effect of Post-operative Radiotherapy on Peripheral Blood Lymphocytes in Patients with Carcinoma of the Breast. *Cancer, N.Y.*, **29**, 349.

MHYER, K. K. (1970) Radiation-induced Lymphocyte-immune Deficiency. *Archs Surg.* **101**, 114.

RAJAPAKSE, D. A. & GLYNN, L. E. (1970) Macrophage Migration Inhibition Test using Guinea-pig Macrophages and Human Lymphocytes. *Nature, Lond.*, **226**, 857.

SEGALL, A., WEILER, O., GENIN, J., LACOUR, J. & LACOUR, F. (1972) *In vitro* Study of Cellular Immunity against Autochthonous Human Cancer. *Int. J. Cancer*, **9**, 417.

SØBORG, M. & BENDIXEN, G. (1967) Human Lymphocyte Migration as a Parameter of Hypersensitivity. *Acta med. scand.*, **181**, 247.

STEWART, T. H. M. & ORIZAGA, M. (1971) The Presence of Delayed Hypersensitivity Reactions in Patients towards Cellular Extracts of their Malignant Tumors. *Cancer, N.Y.*, **28**, 1472.

STJERNSWÄRD, J., JONDAL, M., VANKY, F., WIGZELL, H. & SEALY, R. (1972) Lymphopenia and Changes in Distribution of Human B and T Lymphocytes in Peripheral Blood Induced by Irradiation for Mammary Carcinoma. *Lancet*, i, 1352.

WOLBERG, W. H. (1971) Inhibition of Migration of Human Autogenous and Allogeneic Leukocytes by Extracts of Patients' Cancers. *Cancer Res.*, **31**, 798.

WOLF, A. (1969) The Activity of Cell-free Tumour Fractions in Inducing Immunity across a Weak Histocompatibility Barrier. *Transplantation*, **7**, 49.